# TRP ion channels: Proteins with conformational flexibility

**DOI:** 10.1080/19336950.2019.1626793

**Published:** 2019-06-11

**Authors:** Ana Elena López-Romero, Ileana Hernández-Araiza, Francisco Torres-Quiroz, Luis B. Tovar-Y-Romo, León D. Islas, Tamara Rosenbaum

**Affiliations:** a Departamento de Neurociencia Cognitiva, División Neurociencias, Instituto de Fisiología Celular, Universidad Nacional Autónoma de México, Mexico, Mexico; b Departamento de Bioquímica y Biología Estructural, División Investigación Básica, Instituto de Fisiología Celular, Universidad Nacional Autónoma de México, Mexico City, Mexico; c Departamento de Neuropatología Molecular, División Neurociencias, Instituto de Fisiología Celular, Universidad Nacional Autónoma de México, Mexico City, Mexico.; d Departamento de Fisiología, Facultad de Medicina, Universidad Nacional Autónoma de México, Mexico City, Mexico

**Keywords:** TRP Channels, structure-function, Ion channels, lipid-protein interactions

## Abstract

Ion channels display conformational changes in response to binding of their agonists and antagonists. The study of the relationships between the structure and the function of these proteins has witnessed considerable advances in the last two decades using a combination of techniques, which include electrophysiology, optical approaches (i.e. patch clamp fluorometry, incorporation of non-canonic amino acids, etc.), molecular biology (mutations in different regions of ion channels to determine their role in function) and those that have permitted the resolution of their structures in detail (X-ray crystallography and cryo-electron microscopy). The possibility of making correlations among structural components and functional traits in ion channels has allowed for more refined conclusions on how these proteins work at the molecular level. With the cloning and description of the family of Transient Receptor Potential (TRP) channels, our understanding of several sensory-related processes has also greatly moved forward. The response of these proteins to several agonists, their regulation by signaling pathways as well as by protein-protein and lipid-protein interactions and, in some cases, their biophysical characteristics have been studied thoroughly and, recently, with the resolution of their structures, the field has experienced a new boom. This review article focuses on the conformational changes in the pores, concentrating on some members of the TRP family of ion channels (TRPV and TRPA subfamilies) that result in changes in their single-channel conductances, a phenomenon that may lead to fine-tuning the electrical response to a given agonist in a cell.

## Introduction

The appearance of the plasma membranes of cells constituted a key event for life during evolution. These structures represent boundaries of the units of life, achieving pivotal functions for the cell, including transport of molecules, communication with the environment, and metabolic functions. The intrinsic hydrophobic nature of these structures renders them impermeable to the flux of charged molecules, a process necessary for cell-cell communication, activation of signaling pathways, as well as for the ability to respond to endogenous and exogenous signals. This issue is resolved by the presence of certain types of proteins in plasma membranes, called ion channels that allow for the passage of ions from one side to the other. The specific structure of one ion channel may differ substantially from another; however, all these proteins contain a pore that opens and closes to permit the flow of ions, mediating ionic currents that, in turn, result in the generation of signals with distinct physiological consequences.

There is an intimate relationship between the structures and the functions of ion channels, with their different component regions serving a specific role in the activity of these proteins. In the 1980s, when the patch-clamp recording technique was developed, it became possible to study the behavior of single ion channels in real time[1]. Furthermore, with the advances seen in the areas of molecular biology, cloning and generation of knockout animal models, we have been provided with valuable methods[1] to carefully determine the roles of ion channels in normal and under pathophysiological conditions. A large variety of ion channels have been cloned and biophysically characterized and, in the past three decades, research-oriented towards determining the structure of ion channels has witnessed remarkable progress.

The Nobel Prize in Chemistry was awarded to Roderick MacKinnon and Peter Agre in 2003, for advancing the field by resolving the molecular structures K^+^ channels [2–5] and aquaporins [6,7], allowing us to further understand their functional features.

The present review focuses on a family of proteins named Transient Receptor Potential (TRP) ion channels, which has fundamentally reshaped our knowledge of sensory physiology and their importance is illustrated by their pivotal roles in smell, taste, vision, touch and our ability to detect changes in temperature. Originally, the first *trp* gene was identified in a *Drosophila* mutant with altered vision [8]; however, it was not until it was cloned that the deduced amino acid sequence led to the suggestion that *trp* encoded for a cation channel [9]. Electrophysiological studies later showed that *trp* indeed was an ion channel and that its selectivity could be modified by inserting mutations in the amino acid region that formed the pore loop [10].

The field of study of TRP channels witnessed a boom when mammalian homologs of the *Drosophila trp* channel were cloned and when some of these were identified as temperature sensors [11,12]. A feature of these proteins is that several of these channels are polymodal, that is, being activated by several distinct physical stimuli and more than one ligand. In some cases, different biophysical properties as well as distinct conformational changes in their pores, associated with interactions with different ligands, have been demonstrated.

We will discuss a few examples of ion channels where conformational changes are associated with different ion conductance states. However, we will mainly focus on TRPV1 and other TRP ion channels for which structures have been solved, making emphasis on the conformational changes produced by various ligands and on their effects in the functional properties of these proteins.

## Rearrangements in the outer pore of kv2.1 lead to changes in ion conductance

For voltage-gated ion channels, modulation of macroscopic current magnitude archetypically is thought to occur through an effect on the gating (opening and closing properties) of these proteins. In this sense, most studies of gating have concentrated on the voltage-dependent gate at the cytoplasmic entrance to the pore. Moreover, there is a large body of evidence that also supports an important role for the selectivity filter in gating activation [13,14]. It has been suggested that some form of voltage-dependent gating can exist at the selectivity filter (the region that discriminates among types of ions passing through the pore) [15–17], although the main closed-open transition is controlled by the S6 bundle-crossing intracellular gate [18].

These aspects have been explored in Kv2.1 potassium channels, which are slowly inactivating delayed rectifiers present in non-neuronal excitable cells and several neurons. In these channels, the current magnitude is modulated by the external K^+^ concentration, making outward currents through Kv2.1 channels become larger when the extracellular K^+^ concentration is increased [19]. Kv2.1 channels exhibit two distinct pharmacological profiles as a function of the K^+^ concentration since they can either be sensitive to external tetraethylammonium (TEA; IC_50_ ~ 3–5 mM), or completely insensitive to this blocker [20]. The underlying mechanism of these effects encompasses the opening of Kv2.1 channels into one of two different outer vestibule conformations with different sensitivities to TEA. It has been shown that the channels that open into a TEA-sensitive conformation produce larger macroscopic currents [19]. In contrast, channels that open into a TEA-insensitive conformation, a phenomenon that occurs in the presence of higher potassium concentrations, yield smaller macroscopic currents [19].

Trapani and collaborators examined the mechanism by which conformational changes in Kv2.1 channels produced changes in current magnitude. By using a combination of single-channel, macroscopic recordings, and hidden Markov modeling they proposed a model in which an outer vestibule lysine residue in position 356 (pore turret region) interferes with the flux of K^+^ through the channel [19]. This led to the suggestion that the K^+^ concentration-dependent change in orientation of that specific K356 can modify single channel conductance through a change in the level of such an interference [19].

In summary, in the conformation where currents are smaller in magnitude, K356 would be oriented towards the center of the ion conduction pathway, not easily allowing the flux of K^+^ ions and resulting in a change in the single-channel conductance. In contrast, when K356 is oriented away from the conduction pathway, it readily allows the passage of ions. Thus, these results obtained by Trapani et al., not only provided evidence where Kv2.1 single-channel conductance is modulated by the outer end of the conduction pathway through the occupancy of open states with different outer vestibule conformations, but they also showed that these occur under physiologically-relevant K^+^ concentrations [19].

Hence, in a physiological scenario, it is important to consider that with rising extracellular K^+^ concentrations, the current magnitude of K^+^ channels in most neuronal cells is reduced as a result of a decrease in electrochemical driving force. However, Kv2.1 counteracts this reduction in net outward current in the presence of higher K^+^ concentrations and, as suggested by the authors, this would provide the cells where these channels are expressed with a mechanism that maintains action potential integrity when high-frequency firing conditions are present [19].

## The validity of the pore dilation phenomenon to explain changes in ion channel conductance

Ion channels exhibit different permeability and selectivity to various ions. Potassium, sodium, and calcium channels contain ion binding sites in their selectivity filters that enable them to finely discriminate among the ions that will flow through their pores^5^[21],. Nonetheless, some ion channels have been shown to exhibit dynamic changes in their ion selectivity in response to agonist activation that could, in theory, allow for changes in their conductance. Examples of these ion channels are P2X receptor channels [22,23], acid-sensing ion channels (ASIC) [24,25] and TRPV1 channels [26,27]. This phenomenon has been called pore dilation and it will be next discussed for P2X receptors, a well-studied example of this process, and for TRPV1 in a later section.

P2X receptors are ion channels gated by extracellular ATP [28] and it was originally thought that the pore first opened rapidly to a conducting state selective for small cations such as Na^+^, K^+^ and Ca [2]+ (I_1_ current) and gradually increased in size or dilated over time, rendering the channel permeable to large organic cations such as N-methyl-D-glucamine (NMDG^+^) or I_2_ current, as well as to large fluorophores and dyes (i.e. YO-PRO1) [22,23]. However, based on the original assumption that P2X receptors experience a slow, time- and agonist-dependent pore dilation, three mechanisms have been suggested to explain this phenomenon: (1) it represents an intrinsic gating property of the functional P2XR channel, where ATP exposure results in the widening of the pore and a change from a state that conducts Na^+^, K^+^, Ca^2+^ (I_1_) to one that conducts NMDG^+^ and YO-PRO1, a large propidium dye. Such a switch has been proposed to be modulated by phosphorylation or dephosphorylation events [22,23]; (2) macropores are formed as a result of an agonist-dependent redistribution and oligomerization of P2XRs. The fusion of two or more trimeric P2XRs, as well as an enlargement of the channel pore, or the formation of a separate but larger pore among aggregating trimeric assemblies, could underly macropore formation. This hypothesis has been ruled out by some research groups since no clustering or redistribution of channels expected during oligomerization has been found to occur [23,29], but some others still support it [30]; (3) the permeability to large cations is facilitated by a structurally separate transport pathway downstream of P2XR activation [22].

Although the phenomenon of pore dilation has been extensively explored, accumulating evidence suggests that it may be an artifact of the experimental conditions used, as will be detailed in the Discussion section.

Next, we will discuss some structural characteristics of TRP ion channels that shed light into why these proteins represent an example of conformational flexibility in response to agonists that may result in changes in cellular excitability.

## The structures of thermotrp channels reveal conformational flexibility

The TRPV1 channel was the first mammalian TRP channel to be cloned. In 1997, it was shown that TRPV1 is the receptor for capsaicin [11,12]. In this groundbreaking study, the laboratory of David Julius isolated a cDNA clone that reconstituted the response to capsaicin in non-neuronal cells. By examining the amino acid sequence of this clone, they demonstrated TRPV1 is an integral membrane protein, homologous to a family of putative store-operated calcium channels and that it is expressed by small-diameter neurons within sensory ganglia such as the dorsal root (DRG) and trigeminal (TG). Moreover, these authors showed that TRPV1 is also a thermal sensor, activated in response to temperatures in a range known to elicit pain-associated behaviors in animals and pain in humans [11]. It was later shown, in 1998, that TRPV1 is also activated by low extracellular pH (pH ≤ 5.9) [12].

Continued work on the study of the function of TRPV1, one of the best studied TRP channels, has led to the identification of several other agonists and modulators including resiniferatoxin (RTx) [12], diacylglycerol [31], hydroperoxyeicosatetraenoic acid (HPETE) [32], anandamide [33], *N*‐acyl ethanolamines (NAEs) [34], n-acyl-dopamines [35], nitric oxide [36], cations [37,38], hydrogen sulphide (H_2_S) [39], the double-knot toxin (DkTx) from the Earth Tiger tarantula [40] and lysophosphatidic acid (LPA) [41].

TRP channels are all tetrameric structures. The structure of TRPV1 had been initially solved in the year of 2008 by the group of Vera Moiseenkova-Bell. These authors obtained a 19 Å resolution cryo-EM structure that showed that TRPV1 exhibits a four-fold symmetry with a large open basket-like domain, formed by the N- and C-termini of the channel as well as a compact transmembrane region [42]. In this first solved structure of TRPV1, the authors described a 150 Å tall ion channel which is divided into a “small” compact region (30% of the total volume) and into a “large” region or the “basket-like” domain (70% of the total mass).

**Figure 1.**
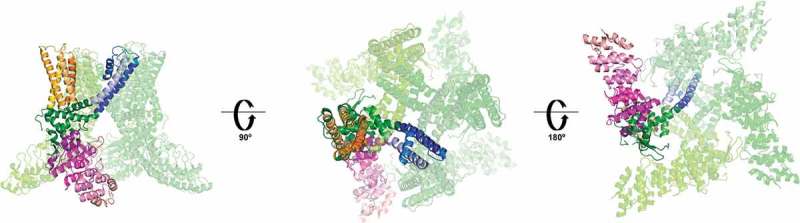
Structure of the TRPV1 channel. Only one of the subunits is highlighted for clarity. The ankyrin repeat domain is shown in purple, the S1- S4 domain in orange, the pore forming S5-P-S6 is shown in blue. The pre-S1, S4-S5 linker, and TRP domain, which participate in the allosteric modulation of the channel, are shown in green. The left panel is a lateral view, the central and right panels are extracellular and intracellular views, respectively. (PDB 3J5P).

This seminal study was followed by the higher resolution structures for TRPV1 obtained by the research groups of Yifan Cheng and David Julius. Two, back-to-back reports, described structures of 3.4–4.2 Å resolution obtained by cryo-EM [43,44]. They confirmed that TRPV1 has a four-fold symmetry with a central ion pathway that is formed by transmembrane segments S5 and S6 and a pore-loop, all of which are surrounded by the S1-S4 domains with a domain-swapped geometry [43,44]. The pore region was described as one containing a wide extracellular mouth and a short selectivity filter. The important TRP domain, which is a region conserved in these family of ion channels and that is thought to play an important role in their allosteric modulation, was shown to interact with the S4-S5 intracellular linker. Each of four subunits in TRPV1 contains six ankyrin repeats that form the ankyrin repeat domain (ARD) localized to their N-termini [43,44] (Figure 1).

**Figure 2.**
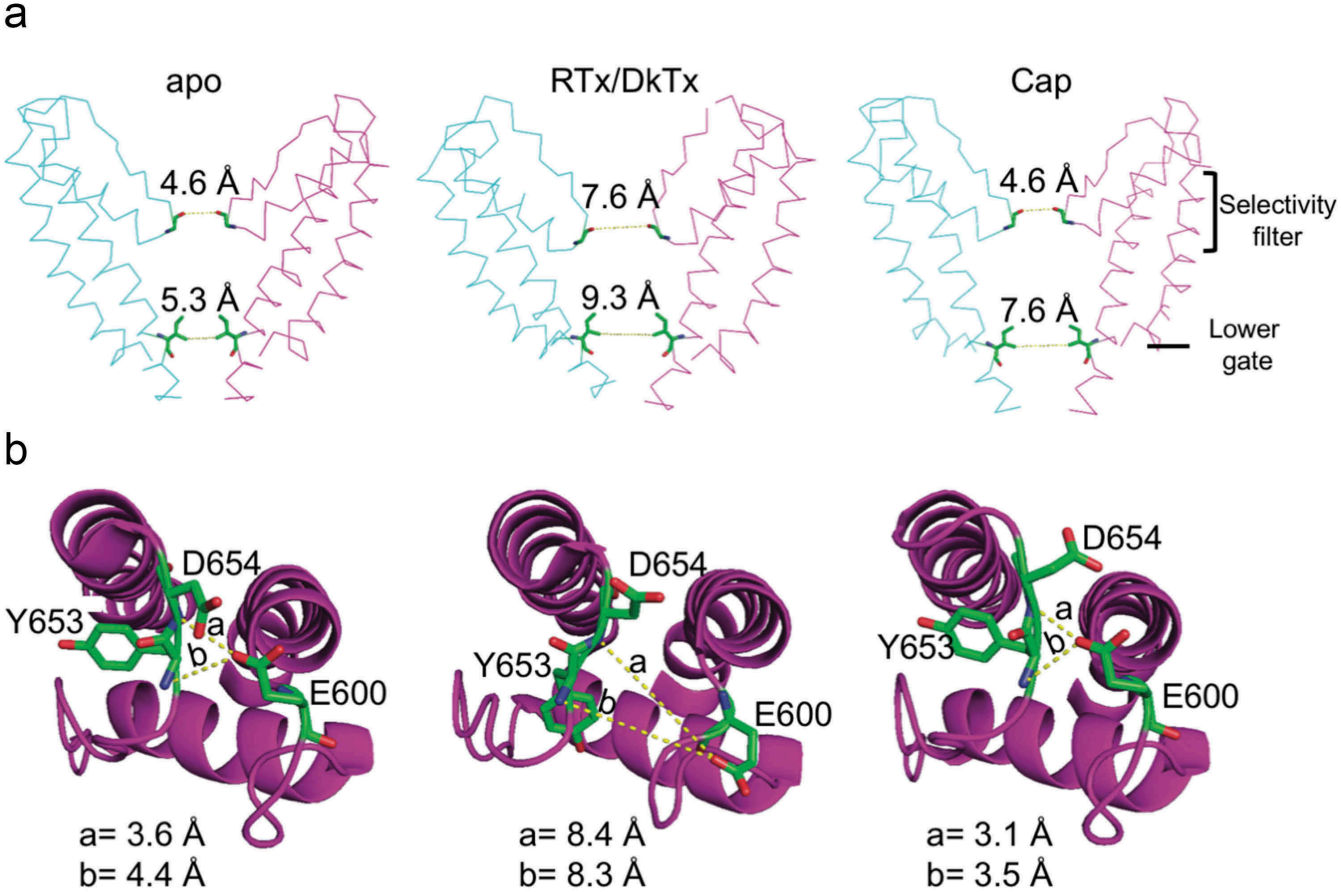
Relative movements of the selectivity filter and lower gate of TRPV1 in the presence of different agonists. **A**. Distances between G643 (at the selectivity filter) and I679 (lower gate) in the apo (left, PDB 3J5P), RTx/DkTx (middle, PDB 3J5Q) and capsaicin (right, PDB 3J5R) structures. Compared to the apo structure, in the presence of both RTk and DkTx the selectivity filter and the lower gate expanded. In the structure with capsaicin only (Cap), the outer pore region remains unaltered in comparison with the apo structure, while the lower gate widens. **B**. Interactions among residues in the outer pore that stabilize the closed conformation of TRPV1. The hydrogen-bonds between E600 side chain with the main-chain nitrogen atoms of Y653 and D654 are broken in the structure with RTk/DkTx but maintained with capsaicin only.

These research groups also described the structures of TRPV1 obtained in the presence of different agonists, providing insight into the mechanisms that allow the polymodal activation of this channel. One of these structures was obtained in the presence of RTx (which also binds to the vanilloid pocket where capsaicin binds through residues Y511 and S512 in S3 and M547 and T550 in S4) and DkTx together. The authors showed that DkTx was bound to the extracellular loops of the outer pore region and defined contacts of the toxin with residues at the top of the pore helix of one subunit and the outer pore loop in the proximal S6 region of another nearby subunit [43,45] (Figure 2).

The structures of TRPV1 obtained in the presence of both agonists display evident rearrangements in the ion conduction pathway and the outer pore region. With RTx and DkTx bound to the channel, the ion conduction pathway appears completely eased of constrictions. This is in contrast to the structure obtained only in the presence of capsaicin, where the lower gate I679 is extended, but the selectivity filter G643 remains unaltered [45] (Figure 2).

When TRPV1 has RTx and DkTx bound, a shift or tilt of the S6 helix 1.9 Å away from the central axis of the channel is produced, as compared to the apo or unliganded state. Moreover, an increase in the distance between the carbonyl oxygens of G643, which constitutes the narrowest point of the selectivity filter, is also observed when both ligands are present: it goes from 4.6 Å in apo (PDB 3J5P) to 7.6 Å in RTx/DkTx (PDB 3J5Q) [45].

As mentioned above, G643 (at the upper gate) remains unaltered between the apo structure (PDB 3J5P) and in the structure in complex with capsaicin, and this could be explained because, in the absence of DkTx, the channel could be undergoing transitions to closed states. However, the distance between the I679 residues (lower gate) in the apo state is 5.3 Å (PDB 3J5P) and, when in complex with RTx/DkTx (PDB 3J5Q), it shifts to 9.3 Å, while it is at 7.6 Å with capsaicin only (PDB 3J5R). Therefore, the authors concluded that the structure obtained with capsaicin is likely only a partially activated state of TRPV1 [45].

When TRPV1 is in the presence of capsaicin only, the structure can be superimposed to that of the apo state in the outer pore region. Hydrogen bond interactions among side chains of the E600 residues and main-chain nitrogen atoms of the Y653 and D654 amino acids in the outer pore loops thought to stabilize the ion channel in the closed state, are broken when the distances among them are increased in response to the binding of DkTx and RTx. The distances between residues that stabilize the closed state change when the channel is in the apo state or in the presence of RTx/DkTx: D654-E600 goes from 3.6 Å to 8.4 Å, while for Y653-E600 it goes from 4.4Å to 8.3Å, respectively [45] (Figure 2).

In summary, this ion channel displays extraordinary flexibility of movement in response to the presence of different agonists.

Another example of conformational changes that may allow for “superactivated” states is that of TRPV2. This channel is activated by temperatures near 52ºC [46] and by membrane stretch [47]. Although structurally similar to TRPV1, TRPV2 is not activated by vanilloid compounds. However, the laboratory of Kenton Swartz produced a TRPV2 channel with four-point mutations to form a vanilloid-binding site located in the S4-S5 linker and the base of S5 [48], rendering this channel sensitive to RTx. These results were also confirmed by Yang et al., who used a TRPV2-Quad channel (with the same point mutations in mouse), where capsaicin competed for the same site as RTx [49]. This TRPV2_Quad channel was found to bind RTx, and that leads the channel to an unstable open state through a mechanism that bridges the S4-S5 linker to the S1-domain [49].

A couple of years later, Zubcevic and collaborators obtained the crystal structure of a minimal construct of the rabbit TRPV2 channel (miTRPV2) in the absence of agonist and compared it with that of the vanilloid-sensitive miTRPV2 (F470S, L505M, L508T, and Q528E, in the rabbit TRPV2) in complex with RTx [50].

**Figure 3.**
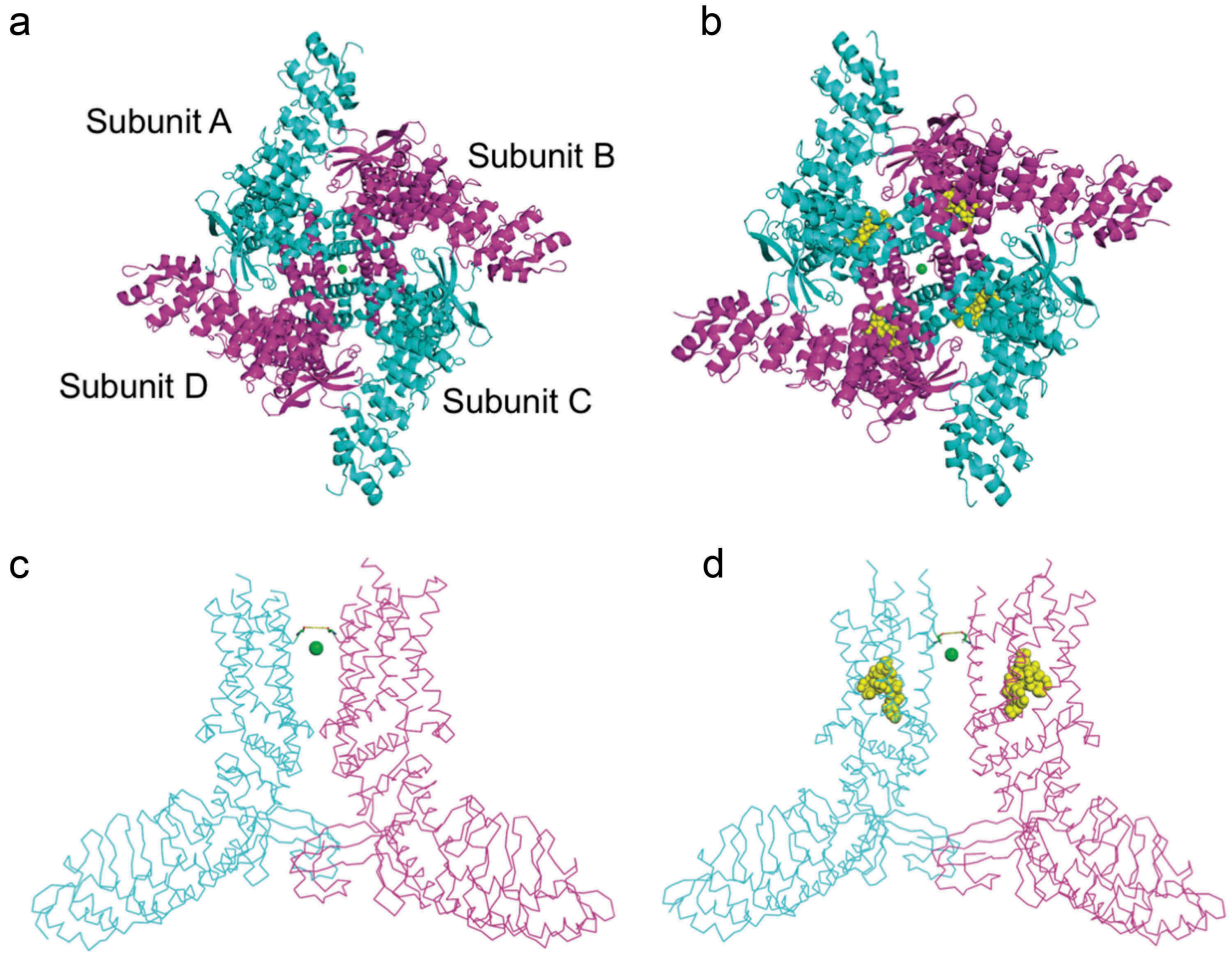
Comparison of open and closed states of TRPV2. Extracellular views of A) a closed miTRPV2 channel (PDB 6BWM) and B) vanilloid-sensitive miTRPV2 (PDB 6BWJ) in complex with RTx shown as yellow spheres, representing the open state. On the lower panel are side views of the closed (C) and open (D) channel. Only two subunits are shown for simplicity. The green sphere is Ca^2+^ trapped in the pore which is notably wider in the open configuration, its also visible the rearrangement of the intracellular domain. The side chains of G604 are also depicted.

Interestingly, in the structure in complex with RTx, they found that two subunits adopt distinct orientations between the S4-S5 linker and the S5, as compared to the other two subunits. In other words, while two subunits (A and C) are found to spread away from each other, the other two (B and D) get closer. This rearrangement results in an asymmetric pore that adopts a wider conformation at the selectivity filter when compared to the structure without RTx (Figure 3).

The authors determined that with RTx the distance between the G604 carbonyl oxygens in contracted subunits was ~7.1 Å, and in the widened subunits the distance between the I603 carbonyls was ~12.3 Å, while in the structure with only Ca^2+^ the distances between G604 carbonyl oxygens are 6.1–6.4 Å [50,51]. The conclusion was that, when RTx binds to the channel, it pushes the base of S5 downwards and produces a bend in the S4-S5 linker producing a different conformation that leads to rotation and widening of the entire involved subunit (Figure 3).

The authors also suggested that this wider conformation of the pore could allow for the permeation of larger cations since they performed experiments where it is observed that, upon activation of the vanilloid-sensitive TRPV2 with RTx (250 nM), uptake of the large fluorescent molecule YO-PRO-1 (376 Da) could be attained. With this experiment, they confirmed the functionality of this conformation, and suggested that it was either a fully open state or an intermediate state that occurred before a symmetric open state capable of permeating large organic cations [50].

Just as in TRPV2, a vanilloid-binding site can be introduced into the TRPV3 channel upon the insertion of six residues in the putative vanilloid-pocket: W521Y, H523E, F522S, L557M, A560T, and Q580E, as shown for the mouse TRPV3 channel by Zhang and collaborators [52]. Nonetheless, the insertion of this vanilloid-binding site is not enough to achieve activation of TRPV3 by RTx and the presence of other agonists, such as 2-APB (aminoethoxydiphenyl borate) or heat, is required [52]. These results demonstrate that gating pathways are conserved among the TRPV channels, but they also show that the energetics for activation can dictate their sensitivity to certain stimuli since adding a binding site for an agonist does not necessarily result in the activation of the ion channel [52].

Structurally, the sensitized state of the human TRPV3 undergoes an α-helix to π-helix transition in the S6 which, in turn, disrupts the S4-S5 linker and S6 and the latter bends ~9° away from the pore [53]. In a previous report, the open conformation of the mouse TRPV3 obtained with 2-APB only shows a π-helix in the S6 [54], leading the authors to suggest that the secondary structure transition could be a hallmark for a sensitized channel, in other words, a state that requires less energy to activate [53].

Additionally, Zubcevic and collaborators also observed that during the activation of TRPV3 with 2-APB, the intermediate states display asymmetrical rearrangements that are not present in the apo or sensitized states. The differently observed deviations for the subunits are caused because theπ-helices in the S4-S5 linkers begin at different residues. This is interesting because it accounts for a similar phenomenon as the one described above for the activation of TRPV2 with RTx, which also originates at the S4-S5 linker [50]. Together, these results further support the hypothesis that the gating mechanisms of TRPV channels are conserved and a break in symmetry may be characteristic to these channel’s gating.

The TRPV4 channel was first described as an osmosensor, capable of activating after exposure to extracellular hypotonic conditions [55,56]. Subsequent reports demonstrated that, like other members of the TRPV subfamily, TRPV4 is a heat-sensitive channel activated by temperatures above 27°C [57] and chemical agonists such as phorbol derivates [58] and 5,6-epoxyeicosatrienoic acid, a metabolite of arachidonic acid [59,60].

Deng and collaborators obtained the first high-resolution cryo-EM structure (3.8 Å) of the TRPV4 channel [61]. These authors determined that the channel was in a closed conformation, that the narrowest diameter in the pore (5.3 Å) was located at residue M714, which impeded the permeation of ions. Even in the closed state, TRPV4 displayed a particularly wide selectivity filter: the diameter at G675 in TRPV4 was 10.6 Å, while the diameter at G643 in TRPV1 in complex with RTx/DkTx was 7.6 Å[45]. This evidence suggested that unlike TRPV1, the TRPV4 channel lacks an upper gate [61].

The single-channel conductance of TRPV4, when stimulated with hypotonic solutions, has been reported to be around 310 pS, at positive voltages [55]. However, reports for its single-channel conductance during spontaneous activity is near 88 pS [56], which is near the value (around 98 pS) obtained with other agonists such as 4α-Phorbol 12,13-didecanoate (4αPDD) and with heat (around 105 pS) [58]. Altogether these results suggest that the channel can adopt a different functional state when it is stimulated under hypotonic conditions vs. heat or phorbol, and that differences in the conductance of TRPV4 may be observed with other agonists, but further studies must be performed.

TRPA1 is a channel mainly expressed in nociceptive neurons which responds to multiple noxious stimuli, including pungent chemicals present in garlic, mustard oil, cinnamon or wasabi [62,63]. Moreover, TRPA1 was initially described as a channel activated by noxious cold temperatures [64,65], but there has been controversy around this topic since this channel functions as a temperature receptor in a species-specific fashion [66–68]. Chemical agonists of TRPA1 have been divided based on their mechanism of action into electrophilic and non-electrophilic agonists. Examples of the first are allyl isothiocyanate (AITC) [69] or allicin [62] that activate the channel through covalent modification of cysteines at the N-terminal domain. In fact, allicin can also activate TRPV1 through the same mechanism [70–72]. The non-electrophilic agonists include carvacrol, oleocanthal, Δ9-tetrahydrocannabinol (THC) and acidic pH, which activate TRPA1 by mechanisms not associated with cysteine modifications and which remain mostly unclarified [73–75].

Cavanaugh et al., determined, in excised TRPA1-expressing membrane patches, that the channel needs to be exposed to polyphosphates (PPPi) [76] in order to be activated by AITC, but in this AITC-insensitive conformation (in the absence of PPPi) it can be activated by THC [77]. This result indicates that the channel shifts to a different functional conformation unresponsive to cysteine modification but responsive to activation by a different mechanism [77], each with a distinguishable function and agonist dependence.

The different functional states described by Cavanaugh et al., have not been structurally compared. Unlike TRPV1, the cryo-EM structure for TRPA1 was not obtained in the presence of different agonists, but only with AITC (PDB 3J9P). Nonetheless, it was possible to determine that the pre-S1 helix, linker S4-S5, and TRP-like domain are bound by hydrophobic interactions and represent an important site for allosteric modulation, similar to what happens with TRPV1 [45,78].

Nevertheless, recent studies have compared the structural rearrangements of TRPA1 in the presence of different agonists using techniques such as limited proteolysis combined with mass spectrometry [75]or circular dichroism spectroscopy only for the N- and C- terminal domains [79]. These robust methodologies show important interactions between cytoplasmatic domains involved in the gating of the channel [79], as well as differences between the activation with electrophilic and non-electrophilic agonists [75].

## TRPV5 and TRPV6 display limited structural rearrengements in their pores

Both TRPV5 and TRPV6 ion channels are expressed mainly in the epithelial tissue of the digestive tract and kidney, where they play roles in the reabsorption and transcellular transport of Ca^2+^ [80]. Unlike other members of the TRPV family, TRPV6 and TRPV5 show inward rectification and these highly Ca^2+^-selective proteins are constitutively active under physiological conditions [81]. Despite the high ion selectivity, in the absence of extracellular divalent cations, they can permeate monovalent cations [80].

TRPV5 and TRPV6 channels share structural traits that differentiate them from the other known TRPV channels. Three phenylalanine residues in S6 give rigidity to the pore, and a ring of aspartates in the selectivity filter confers its high Ca^2+^ selectivity [82]. Neutralization mutation of the negative aspartate charge not only affects Ca^2+^ selectivity but reduces inward rectification [80].

Functionally, both channels can be inhibited by endogenous calmodulin (CaM) [83] or by econazole, an antifungal [84], while PIP_2_ helps stabilize their open state [85]. The conformational changes between open and closed states of these channels have been explored by comparing the cryo-EM structures of econazole-bound TRPV5 and of both channels in the presence of either PIP_2_ or CaM [86,87].

One of the main findings of this comparison is that the selectivity filter remains mostly the same regardless of the molecule that binds the channel. The lower gate identified in TRPV5 is formed by F574, M578, and H582 that constrict the pore to a diameter between 4.5 and 5.9 Å in the same bundle crossing configuration observed in other TRP channels. The closing seems to involve a change in the position of the S4-S5 linker and the loop connecting S6 with the TRP domain [86,87].

The binding of PIP_2,_ induce a change in the orientation of the lateral chain of the aspartate in the selectivity filter. This change may be explained by an interaction of the head group of PIP_2_ with R584 in the S6 helix, analogous to what has been observed in TRPV1, which rotates and pulls away from the center of the pore accompanied by movement of the S4-S5 linker [87]. The main difference in the response of TRPV5 and TRPV6 was observed in the CaM-bound structures. Both channels bind to a single CaM-molecule which is capable of physically blocking the pore from the intracellular side, but while TRPV5 shows no conformational changes, TRPV6 shows an α- to a π-helical transition of S6 which tilts the helix toward the center, so I575 becomes an inactivation gate [87], which has also been observed in TRPV3 [53].

Analysis of the pores of TRPV5 and TRPV6 points to rigid structures that contrast with the more flexible pores of TRPV1-4. To date, there is no physiological evidence of changes in their single-channel conductances in response to different stimuli, but it has been suggested that their activity might be regulated by the formation of TRPV5/V6 heterotetramers, as they are co-expressed in several tissues. Hoenderop et al. used *Xenopus laevis* oocytes to express such heterotetramers and found that they have Ca [2]+ -dependent inactivation and block by ruthenium red with features intermediate to those of TRPV5 and TRPV6 [88]. Although this is an interesting possibility, they have not been identified *in vivo*, and their biophysical properties at the level of single-channels have not been investigated.

## Lysophosphatidic acid: A pain-producing phospholipid

Another activator of TRPV1 shown by our group to directly interact with this ion channel is lysophosphatidic acid (LPA) [41]. This is the only example for which a change in single channel conductance in a TRP channel has been shown to depend upon the presence of different agonists; thus, we will discuss this phenomenon in detail.

LPA has been extensively linked to the generation of chronic neuropathic pain through its interactions with G protein-coupled receptors (GPCRs) [89–92] and to modulate the activity of several ion channels [93–97]. Regulation of the activity of TRPV1 by molecules of a lipidic nature has been explored, and it has been described that PIP_2_ [98–106], polyunsaturated fatty acids (PUFAs) [107], monounsaturated fatty acids [108] and cholesterol [109–111] can modulate the activity of TRPV1, either directly or indirectly. However, reports of direct interactions of LPA with ion channels, in general, are scarce [95,112,113].

LPA is a phospholipid constituted by an acyl chain of saturated or unsaturated fatty acids associated with a glycerol backbone by ester links, and that contains a phosphate head group [114]. LPA can be naturally found as different species that vary in their acyl-chain length as well as in their saturation (16:0; 18:0; 16:1, 18:1; 18:2 and 20:4).

Our group studied whether LPA could produce acute pain in mice and showed that it is considerably dependent upon TRPV1. These results led us to determine that LPA could, in fact, produce currents and action potentials in DRG neurons from wild-type mice but not in DRG neurons from Trpv1^−/−^animals. Using a heterologous expression system of HEK293 cells transfected with TRPV1, we found that currents could be induced when LPA was applied to the intracellular or extracellular faces of the ion channels and that the responses were dose-dependent [41].

In our studies, we ruled out the participation of GPCR-related signaling pathways on TRPV1 activation by LPA and identified a site of interaction for this phospholipid, residue K710 in the C-terminus of TRPV1 [41]. Confirmation of these results was obtained later by another group in a study where TRPV1 was expressed in lipid bilayers and activated by LPA [115].

Hence, we extended the aforementioned study and determined that, for fatty acids to activate TRPV1, the following features had to be met by these molecules: the presence of a charged phosphate group, a length of, at least 18 carbons of the acyl chain and a mono unsaturation [116].

## LPA produces a different open conformational state to that of capsaicin

Our first study on the effects of LPA on TRPV1 was published in 2011 [41], and since then, we have been studying the specifics of how this molecule regulates the activity of TRPV1. Our most recent study on this subject provides functional evidence that LPA produces a different open conformational state of TRPV1 that has a larger conductance.

In preliminary experiments, we had found that addition of LPA (5 μM) to excised membrane patches of TRPV1-expressing HEK293 cells resulted in larger magnitude macroscopic currents, as compared to those obtained in the presence of saturating capsaicin concentrations (4 μM). We had also observed that LPA (5 μM) produced activation of TRPV1 single-channel currents with an open probability (Po is 0.8) similar to that of capsaicin (4 μM).

These data could be explained as 1) LPA either producing an increase in the number of ion channels in the excised membrane patches (which was highly improbable); 2) An increase in the membrane negative surface charge near the channel leading to an accumulation of positively-charged ions near the pore mouth of TRPV1, 3) Producing a phenomenon termed “pore-dilation” in which the permeability to large ions occurs in the presence of prolonged exposures to agonists and that had only been described for activation with capsaicin [26] and/or 4) a change in the single-channel conductance.

**Figure 4.**
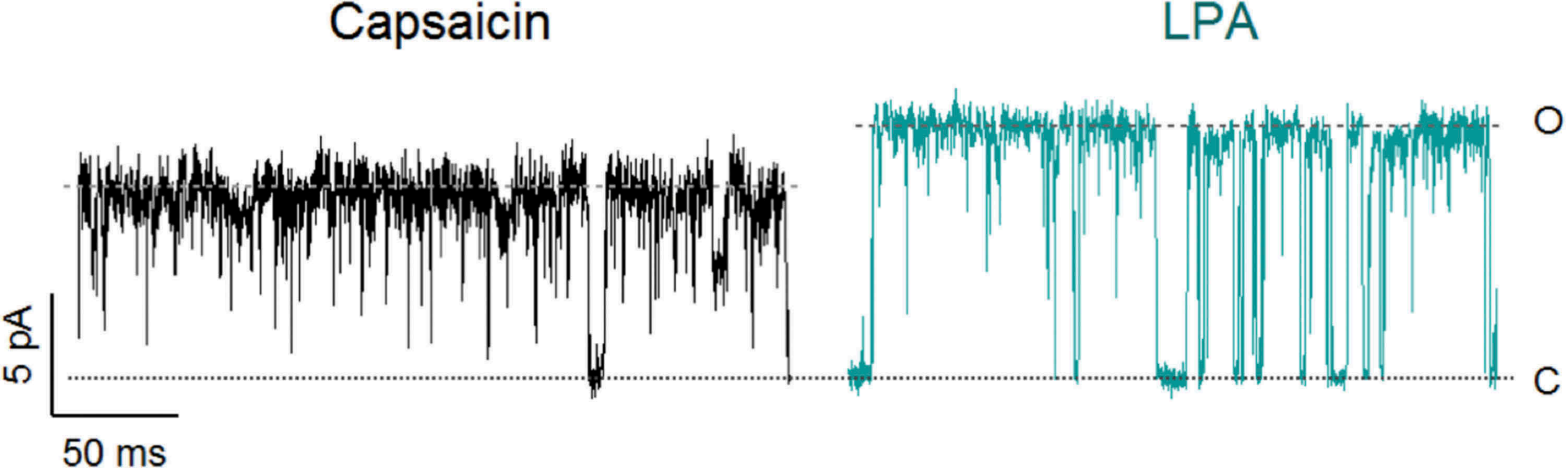
LPA increases single channel currents. Current amplitude for a TRPV1-expressing membrane patch in the presence of capsaicin (4 µM; left) and in the presence of LPA (5 µM; right). Traces were obtained at +60 mV.

In this last study, we performed a set of experiments in order to discern among all of these possibilities. We started by studying how LPA affected the unitary TRPV1 currents by first applying capsaicin (4 μM), washing off the agonist and then applying LPA (5 μM) to the same membrane patch. The results showed that LPA produced a 41% increase in the magnitude of single-channel currents at a single voltage (60 mV), which was also observed at other voltages (Figure 4).

Furthermore, our experiments ruled out all of the other possibilities enlisted above and showed that coapplication of subsaturating concentrations of both agonists together resulted in two different types of openings in a single channel, with conductances corresponding to those achieved either by adding capsaicin or LPA alone [117]. This was the strongest indication of these two agonists producing distinct open conformations that varied in their conductance. Finally, we defined that the presence of K710, the residue that is pivotal for the activation of TRPV1 by LPA, was also necessary for the generation of the higher-conductance open state [117].

## TRPC and TRPM channels

Until just recently, finely defined structural characteristics of TRPC channels have remained elusive, mainly because of the lack of high-resolution structures. The cryo-EM structures obtained to date are those for the TRPC3, TRPC4, TRPC5 and TRPC6 channels [118–123]. However, no functional studies reporting changes in the conductance of these channels in response to different agonists are available.

These channels exhibit four ankyrin repeats and linker helices at their N-terminal domains and at the C-terminal domains they possess a TRP domain that connects a helix and a coiled-coil domain. Inside the pore, all TRPC members have a conserved “LFW” motif that maintains the structure of the pore [124].

The TRPC subfamily can be divided into two groups that share some distinctive characteristics. The first group is constituted by the TRPC3/C6/C7 channels are activated by DAG. Structurally, both TRPC3 and TRPC6 display a pre-S1 elbow, a TRP re-entrant helix embedded in the membrane bilayer after the TRP domain, and an unusually long S3 that forms a “pseudo” extra cellular domain (ECD) [121,122,124]. Furthermore, unlike most TRP channels, TRPC3 channels possess a linker that connects the TRP domain to the S6. This results in a more flexible TRP domain that exhibits a higher coupling with the S4-S5 linker and a lipid binding site near the pre-S1. All of these peculiar interplays of domains may provide a molecular basis for TRPC3 lipid gating [121].

The second group includes the TRPC4/C5 channels that are modulated by G_q,_ G_i/o_ GPCRs, membrane lipids, and intracellular Ca^2+^ [119,125]. These channels share the common feature of a disulfide bond between two cysteines at the loop linking S5 and the pore domain that maintains the residue E555 in proper position and thus stabilize the upper region of their selectivity filters [119,120,123,124]. As in other TRP channels, the TRP domain of TRPC4 is an important molecular effector for gating in this protein since it interacts with the S4-S5 linker and other proteins [123].

The structures of TRPC3/C6 and TRPC4 (both human and mouse) show a closed pore conformation. For TRPC3 the lower gate is located at residues I658 and L654 where they present a radius of less than 1 Å [121]. As for the human TRPC4, the residues I617 and N621 were identified as the lower gate where the constriction of the diameter was around 1.6 and 0.7 Å (defined by opposing van der Waals surfaces) [123], and for the mouse TRPC4 the narrowest point is located at N621 (3.6 Å) and constitutes the lower gate along with I617 and Q625 [119].

However, the TRPC5 is considered at least partially open since the constriction at the lower gate formed by the residues I621, N625 and Q629 was around 4.9 Å [120]. Comparing this structure with the closed TRPC4, Duan and collaborators suggest that the extracellular disulfide bond is a transducer of conformational changes. Nonetheless, more structures of TRPC channels at their open conformation will help to elucidate if there are differences in the pore during their gating [120].

The TRPM subfamily is composed of eight members with diverse features and functions [126]. The TRPM members for which cryo-EM structures have been solved in the closed or partially open conformations are TRPM2, TRPM4, TRPM7 and TRPM8 [127–135]. Their subunits are composed of six transmembranal segments, but at their N-terminal domains, they present four TRPM homology regions (MHR) and a C-terminal coiled-coil domain [127–135].

The C-termini of TRPM2, TRPM6, and TRPM7 channels possess enzymatic domains and have been named “chanzymes” [136–139]. Similar to TRPC4/C5 channels, TRPM channels possess disulfide bonds at their extracellular pore that stabilize the pore domain, but these disulfide-bonds form between cysteines at the pore loop and the pore helix S6 [119].

TRPM2 is a non-selective cation channel sensitive to temperature [140] that is activated when ADP-Ribose (ADPR) and Ca^2+^ are co-applied [141]. Wang and collaborators obtained the structures of human apo-, ADPR-bound, and ADPR-Ca^2+^-bound TRPM2 channels. They observed that the binding of ADPR alone produced a closed conformation, considering it a “primed state” [128].

The lower gate was identified at residues I1045 and Q1053 in the pore and shown to be enlarged in the ADPR/Ca^2+^ structure. Although a change distance of the residues that form the gate was observed, it was not considered large enough to allow passage of Ca^2+^ (~4 Å radius) [128].

For zebrafish TRPM2 channels it was shown that, once ADPR binds to the MHR1/2 domain, a displacement of MHR4 is transmitted to the TRP helix, pushing it towards the S4-S5 linker. Consequently, the S4-S5 relocates the S5 and S6 and finally produces the opening of the channel. Again, as for other TRP channels, the TRP helix is shown to act as a transducer between the intracellular and transmembranal domains during gating [129]. Moreover, the authors propose putative binding sites for Ca^2+^ in the S3, leading them to speculate that Ca^2+^ facilitates the opening due to the movement of the S3 upon the binding of this ion which allows for the relocation of the S4-S5 linker [129].

TRPM4 and TRPM5 channels are among the only TRP members that are not permeable to divalent cations [132,142,143]. Despite their ion selectivity, TRPM4/5 channels are activated upon intracellular Ca^2+^ increases [144] and can be modulated by PI(4,5)P_2_ [145,146]. However, TRPM4 is inhibited by intracellular ATP and nucleotides, while TRPM5 is insensitive to these molecules [147,148].

The pore of TRPM4 has two gates: one located at the selectivity filter and another formed by the intracellular portion of the S6. In the human channel, the selectivity filter gate is formed by residues 975-FGQ-977 [130–132]. Likewise, the intracellular gate is formed by the I1040 residue [131,132].

Strikingly, both gates remain unchanged in all reported Ca^2+^-bound structures, and it has been suggested that Ca^2+^- binding is required for the opening of the channel in response to voltage [131].

In the structure obtained in complex with ATP, Guo and collaborators located a nucleotide binding domain along with twelve helices and two ankyrin repeats in the N-terminal [149]. The ATP-bound structure is an inhibited state of TRPM4, and there were no overall changes in the pore compared to the apo structure. The TRP domain forms hydrophobic interactions with the S4-S5 linker and forms a cavity between the S1-S4 domain, which harbors a potential Ca^2+^ -binding site [149].

The protein kinase domain in TRPM7 induces phosphorylation of receptor tyrosine kinase (RTK)-signaling intermediates and chromatin modifications [150,151]. TRPM7 is permeable to Mg^2+^, Zn^2+^ and Ca^2+^ [152], and the cryo-EM structure of TRPM7 was obtained in the presence and absence of Mg^2+^ [133].

There were no notable changes on the pore domain of TRPM7 under different ionic conditions. Interestingly, the selectivity filter identified in TRPM7 (1045-FGE-1047), varies only on one residue to the selectivity filter described in TRPM4 (971-FGQ-973 in mouse TRPM4 channel), which is crucial to confer the monovalent selectivity of the latter. The lower gate was determined to be formed by I1093 and N1097 residues [133].

The TRP domain of TRPM7 interacts with different regions of the channel, highlighting the importance of this region for gating in this protein. For example, the TRP domain interacts with the N-terminal residues S744 and Q740, with the S4-S5 linker by forming hydrogen bonds between W1111 and R1115 (both located to the TRP domain) and with A981 and V982 (in the S4-S5 linker) [133]. Additionally, it establishes π-π stacking (Phe1118/Tyr1122) and cation- π interactions (Arg1115/Trp1111) among its α-helical structure [133]. However, unlike what has been observed in other TRP structures, the TRP domain of TRPM7 does not contact the pre-S1 helix [133].

Another region that may produce important conformational rearrangements in TRPM7 is the pore helix. It has been shown that a disulfide-bond established between residues C1056 and C1066, is only formed in the presence of Mg^2+^. Thus, it has been proposed that this cation could stabilize the bond and impact directly on the structure of the pore [133].

Finally, the TRPM8 is a non-selective cationic channel permeable to Ca^2+^ and activated by cold temperatures (below 25°C) and menthol [153]. The channel is modulated by PI(4,5)P_2_ [153,154].

In the same report, the cryo-EM structures for TRPM8 have been obtained in complex with PIP_2_, Ca^2+,^ and icilin, and with WS-12 (a menthol analog) together with PIP_2_ [134]. These structures demonstrate that both, icilin and WS-12, bind to a cavity formed between S1-S4 and the TRP domain [134].

The structures reveal a different binding site of PIP_2_, as compared to other TRP channels. This site is formed by the pre-S1 domain, the S4-S5 junction, the TRP domain and the MHR4 of an adjacent subunit. Interestingly, upon the binding of PIP_2_, the S4 undergoes a change from a complete α-helical to a 3_10_ helical conformation [134]. This produces the movement of the TRP domain and the S5 toward the cavity. At the same time, it disrupts the interaction among the S1-S4 domain and the S6-pore domain, enabling gating [134]. Additionally, during gating, the S1-S4 domain of TRPM8 suffers a rigid rotation away from the pore [134], unlike what happens to the S1-S4 domain in TRPV1 which remains static [45].

The lower gate of TRPM8 is formed by L973 where the tightest constriction along the pore was found in a previous structure^134,135^, Yin and collaborators determined that both structures (PIP_2_-Ca^2+^-icilin and WS-12-PIP_2_) were non-conducting states. Although, in the icilin-PIP_2_-Ca^2+^ structure the S6 is curved, which suggested that there may be a π helical turn along the S6 as previously described for the sensitized state of TRPV3^53^. Therefore, the structures may represent sensitized or presensitized states of TRPM8 [134].

Although conformational changes in TRPM channels in response to agonists or modulators of their activities have been clarified, changes in their conductance levels in response to these molecules have not been reported.

## Discussion

Here we have summarized some phenomena that could potentially lead to changes in the conductance of single ion channels. For example, pore dilation is a phenomenon suggested to occur in TRPV1 [26,155] and other channels such as the P2X receptors, and it is characterized by a change in the permeability of the ion channel to larger ions, induced by long times in the presence of the agonist. However, the results that we have obtained, as well as those of other groups, suggest that permeability changes may result from changes in the cytoskeleton organization or from time-dependent local changes in the concentrations of ions in the vicinity of the channel, a phenomenon known as ion accumulation, that is at play in whole-cell experiments [156] and is further discussed below.

Li and collaborators have examined this carefully and have shown that P2X receptors readily activate and do not display a slow phase of activation under symmetrical ionic conditions [156]. These authors also show that changes in the equilibrium potential are observed with NMDG^+^ in the external solution during sustained activation and that this prolonged activation in bi-ionic solutions primes the depletion of internal Na^+^ and accumulation of NMDG^+^ in the cell. Li et al., also discuss that P2X receptors are exceptional in that they demonstrate significant permeability to large cations such as NMDG^+^ and that, even in the absence of pore dilation, would allow the conduction of relatively large molecules to enter or exit cells [156]. Finally, and most importantly, these authors point out that changes in ion concentration, produced by ion accumulation and revealed during experiments with P2X receptors, are an inherent problem in voltage-clamp recordings, where the flow of ionic currents across the membrane may alter the concentrations of intracellular ions [157,158]. In other words, when symmetrical ionic conditions are used, ion concentrations can be maintained by working under conditions in which current amplitudes are moderate and current measurements will always be equal to or greater than the flux of any individual ionic species. This is in contrast to experiments performed with asymmetric ionic conditions, which require a membrane conductance of an order of magnitude smaller than the access conductance to adequately control ion concentrations.

These results are prompting a reexamination of the concepts of pore dilation and dynamic ion selectivity for all ion channels [156], and highlight the importance of further studying the molecular mechanisms underlying different conformational changes induced by ligands in these proteins.

TRPV1 has been shown to contribute to several physiological and pathophysiological processes, importantly. Among these, the participation of TRPV1 in pain-related processes is an aspect that has attracted the attention of several research groups. Understanding cellular mechanisms leading to the generation of pain constitutes the basis for designing tools in order to attack them. Hence, research directedtoward defining the molecular mechanisms that underlie the production of pain is of invaluable importance. This requires a great effort from many researchers and from several points of view, including that of understanding various particularities of the modulation of the effectors involved in pain processes.

As for changes in the conductance of TRPV1 due to the actions of agonists, it is interesting to note that, it has been recently reported that DkTx diminishes the unitary conductance by 32% of TRPV1, as compared to that observed with capsaicin [159]. DkTx is a molecule composed of two moieties joined by a short linker, which bind in the outer pore region of the channel so that each motif sits at a subunit interface [45]. Moreover, the effect was eliminated when the linker of DkTx was either absent or elongated and also when the pore turret of TRPV1 was removed. The lack of pore turret in the construct used for the cryo-EM of TRPV1 explains why the structure with DkTx/RTx is observed to be fully open. In fact, the unitary conductance of the construct used to obtain the TRPV1 structure with bound DkTx was similar to that obtained with capsaicin [159].

With this, Geron et al. confirmed that DkTx and capsaicin elicit distinct gating mechanisms that are dependent upon the pore turret of TRPV1. They argued that the binding of DkTx directly restricts the movement of the pore helix and consequently the conductance.

In terms of the physiological relevance of LPA’s actions on the conductance of the TRPV1 channel, one can only hypothesize that in the presence of this agonist, as compared to capsaicin, depolarization of the neuron where it is expressed will occur more efficiently. Since the change in the conductance of the channel also occurs at subsaturating concentrations of LPA (i.e., 1 μM), this would mean that such an efficient depolarization could also occur even when lower levels of the phospholipid are present.

**Figure 5.**
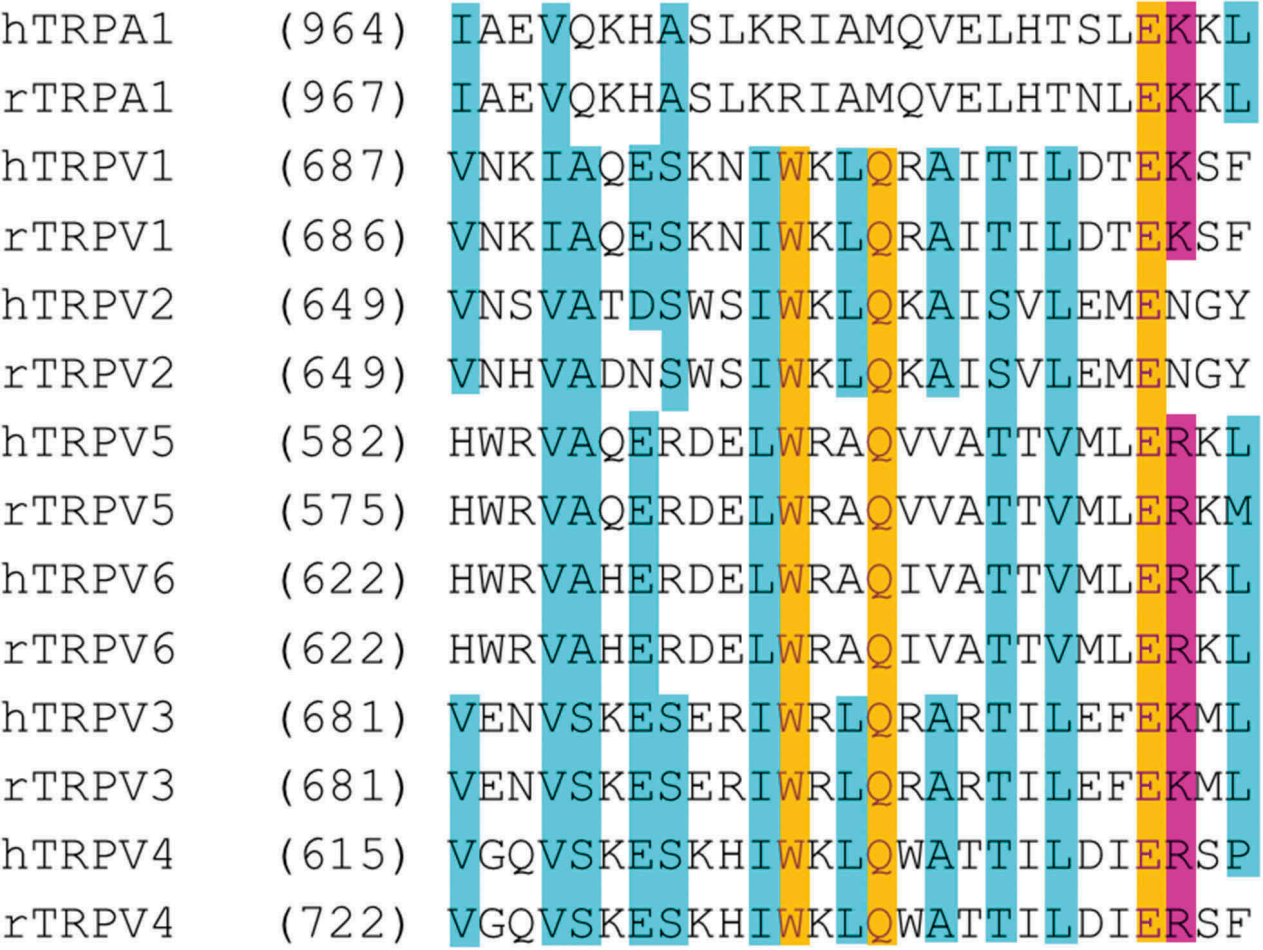
Sequence alignment corresponding to the trp box in rat and human TRPV and TRPA1 channels. Identical residues are highlighted in yellow and similar residues in blue. Tryptophan and glutamine residues in the middle of the trp box are only present in TRPV channels. The residue K710 in hTRPV1 and the corresponding residue in other TRP channels is highlighted in pink, notice that most channels, including TRPA1 have a positively charged residue in that position.

TRP channels share conserved sequences of amino acids in some regions of their structures. One such region is the TRP box (Figure 5), the region where the K710 residue that interacts with LPA lies. We have tested the effects of LPA on some TRP ion channels and have found that TRPV2, TRPV3, or TRPA1 channels are not activated by this phospholipid [41]. Although TRPV3 and TRPA1 have a lysine in this position, just as TRPV1, LPA is not capable of activating these channels. Thus, this phospholipid seems incapable of providing the energy necessary to drive the transition from the closed to the open state in this particular ion channels and the question of why this occurs remains open. Moreover, since other members of the TRPV subfamily of ion channels exhibit a lysine in the corresponding position to that of K710, it will be interesting to test whether their activities can be modulated by LPA. If so, this would constitute the identification of an agonist for these other ion channels for which there are few endogenous activators known (i.e., TRPV4, TRPV5, and TRPV6).

Since achieving different conductance states can modulate the response of the cell where ion channels are expressed, not only will it be interesting to determine if LPA activates these other TRP channels, but also whether it can produce different conformational changes as what happens with TRPV1. This would further demonstrate the conformational versatility of this family of ion channels.

Several ion channels have been studied at great detail with respect to their responses to different agonists. In particular, the idea that LPA produces a conformational change that results in a different conductance of TRPV1 at the single-channel level compared to capsaicin challenges the notion in the field of study of ion channels in general.

Here we have discussed that LPA leads the TRPV1 ion channel to open with a single-channel conductance level that is higher than that attained with capsaicin. Both ligands promote nearly maximal open probabilities [117]; hence, this is most probably not a mechanism in which different subconductance levels can be attained in the presence of partial and full agonists.

Thus, it will be interesting to reflect upon and determine what mechanisms or structural rearrangements allow TRPV1, and maybe other TRP channels, to exhibit a higher conductance level in the presence of one agonist or another.
